# cpCST: a new continuous performance test for high-precision assessment of attention across the lifespan

**DOI:** 10.3389/fpsyg.2025.1640417

**Published:** 2025-09-23

**Authors:** Anna MacKay-Brandt, Daniel Garcia-Barnett, Kai Xuan Gan, Olivia Ripley, Elaine Gazes, Michael Milham, Stan Colcombe

**Affiliations:** ^1^Brain Aging and Mental Health Laboratory, Clinical Research, Nathan Kline Institute, Orangeburg, NY, United States; ^2^Design Acquisition and Neuromodulation Laboratories, Center for Biomedical Imaging and Neuromodulation, Nathan Kline Institute, Orangeburg, NY, United States; ^3^Child Mind Institute, New York, NY, United States; ^4^Columbia MR Research Center, Columbia University, New York, NY, United States; ^5^Department of Psychiatry, New York University Medical Center, New York, NY, United States

**Keywords:** sustained attention, sensorimotor integration, reaction time, behavioral assessment, lifespan development, adaptive testing, task reliability, individual differences

## Abstract

**Introduction:**

Assessing sustained attention presents methodological challenges, particularly when spanning diverse populations whose baseline sensorimotor functioning may vary significantly.

**Methods:**

This study introduces the Continuous Performance Critical Stability Task (cpCST), a novel paradigm combining high-density sampling of behavior (30 Hz), individualized calibration, and fixed-difficulty assessment to measure attentional control. In a sample of 166 adults (ages 18–76), we evaluated the psychometric properties of the cpCST’s instantaneous reaction time (iRT) metric derived through dynamic time warping.

**Results:**

The cpCST demonstrated exceptional reliability (bootstrap split-half r = 0.999) and predictive validity for cognitive performance (flanker and Woodcock-Johnson) and cardiorespiratory fitness (VO2submax). The task achieved high temporal efficiency, with just 2 min of data correlating at r = 0.94 with full-task performance, outperforming a standard arrow-based flanker task. The cpCST’s individualized calibration effectively isolated attentional control processes from baseline sensorimotor function, eliminating age-related slowing effects typically observed in reaction time tasks.

**Discussion:**

This approach offers methodological advantages for lifespan studies, clinical populations, integration with neurophysiological measures, and computational modeling approaches while addressing limitations of existing attention assessment paradigms.

## 1 Introduction

Attention is an intuitive concept that is considered a core component of cognition and everyday functioning (Baddeley, 1996; Duncan, 1986; Norman and Shallice, 1986; Posner and Petersen, 1990), exhibiting a clear trajectory of early life maturation and later-life decline (Fortenbaugh et al., 2015; Tipper et al., 1989). Physiological factors such as fitness are well-known to have a general influence on cognition (Colcombe and Kramer, 2003) and are thought to impact performance through improvements in attentional control (Colcombe et al., 2004, 2006; Prakash et al., 2007). Across the lifespan, attentional processes are linked to the successful navigation of a host of everyday behaviors (Barriga et al., 2002; Bogdanova et al., 2016; Gross, 2015; Halperin, 1991; Kinsella, 1998; Racer and Dishion, 2012; Stierwalt and Murray, 2002); and like many apparently simple behaviors, it can be challenging to define and measure (Anderson, 2021; Shi et al., 2019; Unsworth et al., 2024; von Bastian et al., 2020; Yangüez et al., 2024).

Experimental and clinical work focused on the measurement of sustained attention has produced a wide selection of continuous performance tasks (e.g., Continuous Performance Test: CPT (Beck et al., 1956); AX-CPT (Servan-Schreiber et al., 1996); Psychomotor Vigilance Test: PVT (Dinges and Powell, 1985); Paced Auditory Serial Attention Test: PASAT (Gronwall, 1977); Test of Variables of Attention: TOVA (Leark et al., 1997); Mackworth Clock Test (Mackworth, 1948); Sustained Attention to Response Test: SART (Robertson et al., 1997); Cambridge Neuropsychological Test Automated Battery: CANTAB (Sahakian and Owen, 1992); Continuous Visual Attention Test: CVAT (Schmidt et al., 2024); Gradual-onset Continuous Performance Test: GradCPT (Rosenberg et al., 2013). These tasks are tuned to capture behavioral features thought to contribute to successful performance or identify specific areas of deficit, based on the paradigm and study population of interest (Conners et al., 2003; Cooper et al., 2017; Homack and Riccio, 2006; Klee and Garfinkel, 1983; Lopez-Garcia et al., 2016). Given the complexity of attentional processes and limitations inherent in any one particular paradigm, the development of a large corpus of measures with different features and tuning will facilitate continued knowledge building and translation to practical applications.

As outlined below, opportunities exist to augment or improve upon existing paradigms through novel behavioral sampling, dynamically adaptive assessment, and task calibration approaches - amongst others. Here we describe the Continuous Performance Critical Stability Task (cpCST), which modifies an established sensorimotor integration task to create a novel attention task featuring high-density behavioral sampling, dynamic adaptation, and effective behavioral calibration across the lifespan. We first describe these new features, not readily available in current paradigms, and the proposed advantages of these enhancements. We then present a preliminary psychometric evaluation of the cpCST’s primary outcome metric (instantaneous reaction time; iRT). We also examine predictive validity of the cpCST iRT to flanker task performance, Woodcock-Johnson Intellectual Ability and Achievement scores, as well as a measure of cardiorespiratory fitness (VO2max), before discussing the advantages of the cpCST paradigm in relating physiological and brain timeseries to participant behavior.

In most continuous performance tests, attention is probed via button press responses at discrete intervals ranging from roughly one to several (10+) seconds apart c.f. (DiFrancesco et al., 2019; Dinges and Powell, 1985; Homack and Riccio, 2006); attentional lapses are inferred on the occasion of delayed, missed, or incorrect responses. Despite the relatively sparse sampling of behavior (< 1 Hz - once every second or longer), these response time studies have demonstrated attentional fluctuations over time (Castellanos et al., 2005; Decker et al., 2023; Di Martino et al., 2008; Esterman et al., 2013; Jackson and Balota, 2012; Smallwood et al., 2004); however, higher density sampling of behavior may more effectively characterize the maintenance of focus over time, moment-to-moment fluctuations, and/or lapses in attention. While many established tasks require continuous monitoring of stimuli (e.g., CPT and PVT), they do not sample behavior continuously. Our primary goal for the development of the cpCST was to create a task that sampled behavior at a much higher rate (30 times per second) than existing tests. Further, unlike a stop-signal (Logan and Cowan, 1984) or gradual-onset continuous performance test (Rosenberg et al., 2013), the cpCST does not include the feature of building up a prepotent response as the result of higher frequency responding. This allows for the assessment of continuous attention under qualitatively different conditions than many tests with higher responding rates.

Administering reaction time tasks to older adults, children, or clinical populations often requires adjustments to various parameters such as stimulus type, stimulus modality (audio vs. visual), presentation and response durations, response interval, interstimulus intervals, stimulus set sizes, or proportion of trial types (see Cowan et al., 2010; Craik, 1986; de Souza Almeida et al., 2021; Reuter-Lorenz and Cappell, 2008; Rueda et al., 2004). These adjustments are motivated by group differences in sensorimotor speed, working memory, auditory or visual acuity, etc., (Cerella, 1990; Conway et al., 2003; Denckla, 1996; Jacobson et al., 2011; Kail, 1991; Owsley, 2016; Salthouse, 1996; Verhaeghen and Cerella, 2008; Wingfield et al., 2005). While accommodations such as these allow for versions of standard neurocognitive tasks to be applied across a wider range of individuals, they raise concerns regarding the comparability of results across test variants (Best and Miller, 2010; Cooper et al., 2017; Hedge et al., 2018; Parsons et al., 2019). Additionally, these changes are applied under the assumption that the altered parameters are uniformly appropriate to the group in question [e.g., trading arrow shapes for cartoon fishes in the ANT-C task or an increase in presentation duration for older adults (Gershon et al., 2010; Rueda et al., 2004)], despite well-documented heterogeneity within groups (Fair et al., 2012; Lindenberger and Baltes, 1997; Logan et al., 2023). As part of the NKI-RS2 lifespan characterization study, our goal was to develop a test that did not require different versions across the ages 9–75 years. Our focus was to use simple stimuli and an intuitive response modality to decrease instructional or proficiency barriers. Further we adopted a closed-loop system developed for a sensorimotor paradigm (Jex et al., 1966), described in detail below, that is calibrated to the individual’s own motor performance to equate individual performance differences into a uniform task design. We are not aware of any other continuous performance tasks that incorporate this design feature.

Rather than assuming that a single set of task adaptations will be equally appropriate across a given group (e.g., older adults or children), fully adaptive paradigms individualize task parameters for each participant by dynamically altering key task features in response to ongoing task behavior. Some tasks are explicitly designed to be adaptive [e.g., Stop-Signal Reaction Time (Logan and Cowan, 1984)]. More recent approaches overlay adaptive procedures that alter task features such as presentation time, response windows, or set size, in response to participant performance in real time as the task evolves (Barbey et al., 2022; Draheim et al., 2024, 2021; Schneiders et al., 2011). As such, each participant’s task is custom tailored to their individual performance on that task through approaches such as staircase or Bayesian-based adaptive algorithms (Farahbakhsh et al., 2019; Manning et al., 2018). These approaches are more efficient (Attarha et al., 2024; Davis et al., 2002; Gibbons et al., 2024; Sorrel et al., 2020), and can be leveraged not only in assessment, but also training protocols (e.g., Roheger et al., 2020). However, they also suffer from drawbacks such as edge case and small sample size failures, induction of artifactual oscillatory “yo-yo” patterns in difficulty, as well as the additional complexity involved in dynamically adapting task parameters in real time (García-Pérez, 2011; Kontsevich and Tyler, 1999; Treutwein, 1995).

One promising approach is to leverage the best of both dynamic adaptive approaches and fixed stable approaches. Participant ability is assessed via adaptive staircase or Bayesian methods in a calibration phase. During the test phase, difficulty is set to a fixed level matching the participant’s individual ability (e.g., the stimulus onset asynchrony that resulted in > 70% accuracy) (Chen et al., 2017; Fleming et al., 2010; Lindfield et al., 1994). Under this approach, difficulty is individually tuned - thus avoiding assumptions about the appropriateness or comparability of group-specific stimulus changes. And the difficulty is fixed during the testing phase, reducing computational complexity and artifactual issues such as induced oscillatory behavior or algorithmic failure. For the cpCST, we leveraged such a hybrid approach by incorporating a calibration phase and a test phase. The goal was to maximize our ability to individually calibrate performance on the sensorimotor component of the task so that performance adjustments related to drifting attention could be better compared across individuals who differ in performance on the sensorimotor integration dimension of the task.

The cpCST design is intended to capture attentional dynamics from moment to moment using a simple, relatively short duration, high information density, individualized difficulty approach in order to maximize the detection of attentional control performance differences across a lifespan sample. The cpCST uses the hybrid-calibrated approach to first assess visuomotor ability, replicating the Critical Stability Task developed by Jex et al. (1966), and then employs a fixed difficulty visuomotor continuous performance task based on the individual’s motor stability threshold (MST; see below). Thus the cpCST retains Jex et al.’s (1966) Critical Stability Task name and adds a new continuous performance phase. The cpCST additionally possesses useful features such as simple task instruction and continuous sampling of behavior (@30 Hz; i.e., 30 times per second), providing a robust complement to existing neurocognitive tools.

**FIGURE 1:**
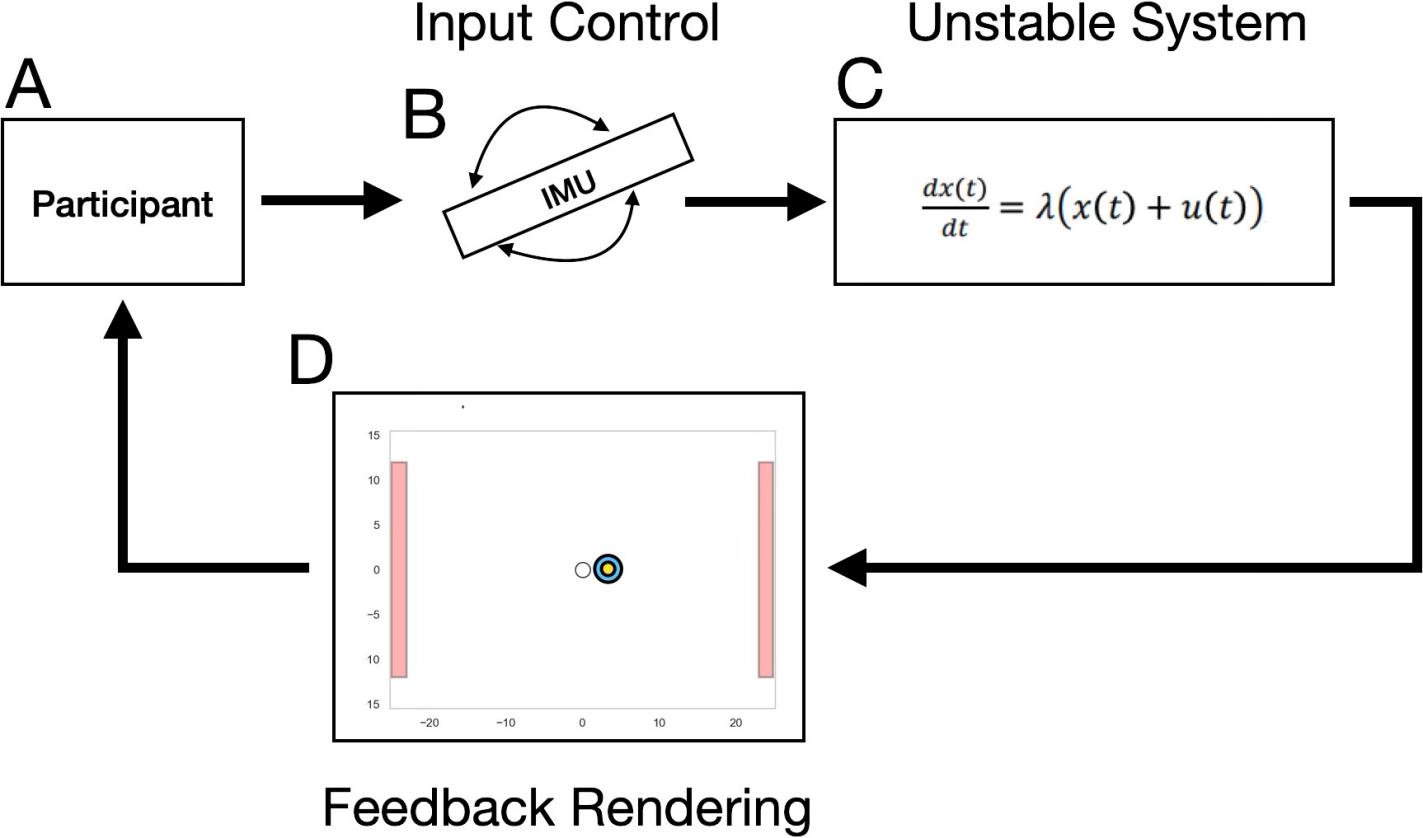
Continuous Performance Critical Stability Task (cpCST) control system: the cpCST is driven by a closed loop feedback system, essentially replicating the approach of Jex et al. (1966), and more recently, Quick et al. (2018). The participant **(A)** is tasked with keeping the stimulus disk at the center of the screen. They controlled the position of the central dot using a handheld controller enabled with an inertial measurement unit **(B)**. To control the movement of the central stimulus disk, participants were required to counteract the drift of the central stimulus by tilting the inertial motion unit (IMU)-enable controller in the opposite direction. The interaction between the stimulus disk movements and participant movements drove an unstable system governed by the equation in box **(C)**, where x(t) represents the horizontal position of the central stimulus disk at each time point, u(t) is the corresponding horizontal position of the participant’s (invisible) cursor. Lambda operates as a gain mechanism on the system, controlling the magnitude with which the discrepancy between participant and stimulus disk positions impact cursor position. Participants are provided visual feedback regarding the current position of the stimulus disk on the computer screen **(D)**.

The cpCST is an extension of a psychomotor tracking task (Critical Stability Task; CST) developed for NASA by Jex et al. (1966) to evaluate pilot performance under unstable control conditions. This simple and elegant foundational paradigm has subsequently been adopted by human and non-human primate laboratories to develop and refine human-machine interfaces (Quick et al., 2018), understand the neural mechanisms of sensorimotor coordination (Sadeghi et al., 2024), and examine drug induced motor control disruption (Ramaekers et al., 2006). We propose that the CST also provides a strong foundation upon which to build a continuous self-calibrated task to assess dynamic fluctuations in attentional control. Specifically, we employed a variant of the original CST to serve as a calibration phase that established a participant’s individual MST. We then used that individualized score to set the difficulty level for that participant’s continuous performance phase. The goal for this new continuous performance phase was to maintain attention on a relatively easy task (set as 30% of MST, based on internal pilot testing) for a 10 min period to capture attentional drift and the latency to respond to a drifting stimulus. It may be useful to clarify that there are a number of continuous tracking tasks in which participants use devices such as joysticks, trackballs, etc., to align a cursor with a spatially moving target item (Colino et al., 2017; Ewolds et al., 2017; Huang et al., 2005; Snow et al., 2016; Yang et al., 2021). The main difference between these sorts of continuous tasks and the cpCST is that the cpCST is based on Jex et al.’s (1966) closed-loop paradigm in which the goal is to maintain the target stimulus at the center of the screen, rather than track, for example, a vertically oscillating target. Additionally, in both the Jex et al. (1966) task and the cpCST, the target’s stability is solely dependent on the user’s movements – there is no externally driven movement of a target object for the user to track. See Figure 1, described in more detail below.

To evaluate this novel task, we embedded the cpCST within the Nathan Kline Institute Rockland Sample II (NKI-RS2), a large-scale, community-based lifespan study. The NKI-RS2 was designed to support the development and validation of next-generation tools for phenotyping normative brain-behavioral associations and investigate the underlying neural and physiological mechanisms that promote mental health across the lifespan. This context offered an opportunity to examine individual differences in attentional control across a wide age range using a task that prioritizes continuous behavioral sampling, individualized calibration, and high-density data collection. We characterized cpCST performance in relation to broader indices of cognitive function and health. In this preliminary analysis, we report behavioral data from the cpCST from a subset of participants to describe the development of a key task performance metric (instantaneous reaction time; iRT) and establish its reliability and preliminary predictive validity on cognitive and physiological indices.

## 2 Materials and methods

### 2.1 Participants

Participants were recruited into the Nathan Kline Institute Rockland Sample II study (NKI-RS2) through prior participation in the NKI-RS research program (Nooner et al., 2012), community outreach, and word-of-mouth. The lifespan sample recruited participants from age 9 to 76 years who were residents of Rockland, Orange, Bergen, or Westchester counties in the north suburban New York City area. All were fluent in English and had no severe physical or sensory limitations, contraindications for MRI or cardiovascular fitness testing, or acute psychiatric symptoms. Participants were excluded if they had a history of schizophrenia, schizoaffective disorder, autism spectrum disorder, or serious neurological conditions (e.g., Parkinson’s disease, traumatic brain injury, dementia). Current psychotropic medication use and serious medical conditions or metabolic disorders affecting the central nervous system (e.g., malignancy, HIV) were also exclusionary. For this preliminary analysis, we included a subset of 166 participants aged 18 to 76 years (M = 51.61, SD = 16.36), 66% female, with complete and quality controlled data for the cpCST and cardiorespiratory fitness procedure. Please note, this analysis is based on a convenience sample extracted from the ongoing NKI-RS2 characterization study to present preliminary findings and introduce novel task development.

### 2.2 Procedures

Sample characterization data were collected via remotely administered surveys on the MindLogger Platform (Klein et al., 2020) and in-person testing. Demographic data was collected via Mindlogger surveys, clinical characterizations were conducted by research staff in-person and via telephone interviews; all cognitive and cardiorespiratory fitness data were collected onsite. The study was approved by the NKI Institutional Review Board, and all participants provided informed consent before undergoing any procedures.

### 2.3 Measures

#### 2.3.1 Continuous Performance Critical Stability Task (cpCST)

The Continuous Performance Critical Stability Task (cpCST) was administered in a dedicated testing room at the Center for Biomedical Imaging and Neuromodulation (CBIN) at NKI. Participants were seated in front of a 61 × 36 cm computer monitor, at a distance of 65 cm. The monitor displayed a circular stimulus at the center of the screen subtending 3.17 degrees of visual angle (DVA). Screen resolution was 1,920 × 1,080 pixels. They were instructed to maintain the position of a circular stimulus at the center of the screen. The stimulus could move along one dimension (left-right on the x-axis). Participants controlled the position of the central stimulus by tilting a custom-built handheld inertial motion unit (IMU) that measured rotation along the x-axis to the left or right. Participants were given a brief (∼2 min) practice round in which they gained familiarity with the controls at a very low difficulty level prior to beginning the calibration phase.

**FIGURE 2:**
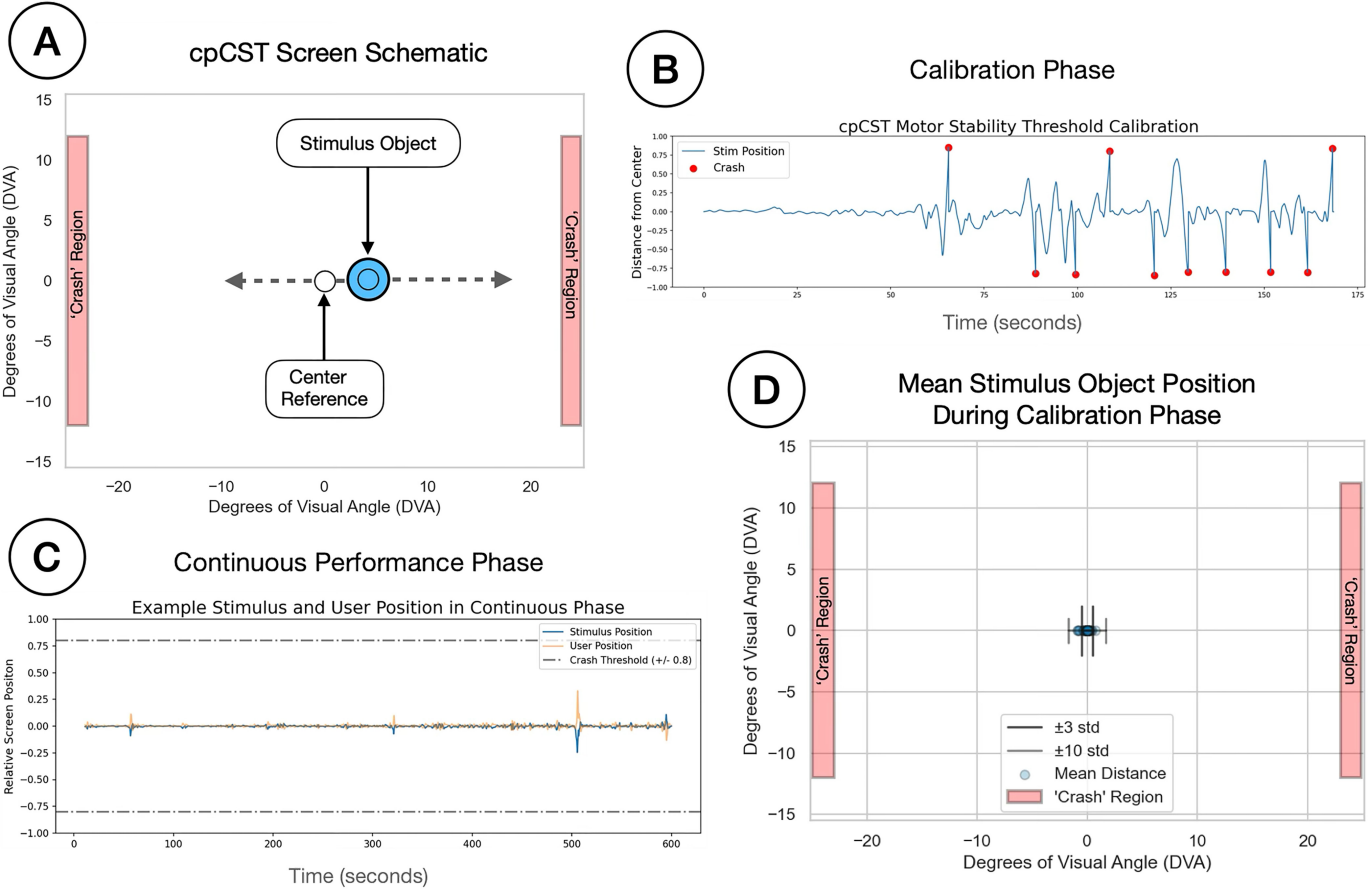
**(A)** Provides a schematic for the Continuous Performance Critical Stability Task (cpCST) task screen. The stimulus object (SO) is a circle that can move in the x (left-right) dimension; participants are asked to maintain the position of the central stimulus at the center of the screen by tilting a custom accelerometer-enabled button box. The screen also contains a central reference point to provide participants with a spatial anchor during task performance, and a pair of crash region rectangles marking the out of bounds point for the SO. **(B)** Shows a time series from the SO position during a cpCST calibration phase, with time (seconds) on the x-axis and stimulus eccentricity on the y-axis. In the calibration phase, participants attempted to keep the SO at the center of the screen as difficulty (lambda) linearly increased until the participant lost control. **(B)** Shows the trace of an SO during the calibration phase (blue line), as well as crashes (red dots), where the SO eccentricity exceeded ± 80% of the distance from the center to the edge of the screen. Participants perform the task through 10 crashes, and the difficulty at the time of the crash is recorded. The user’s motor stability threshold (MST) is calculated by the mean lambda value for the last three crashes. The MST value is then used to set the difficulty for the continuous performance phase. **(C)** Shows the path of the SO (blue line) and the participant tracking position (orange line) when controlling the SO during the continuous phase. Dashed gray lines show the ± 80% crash boundary threshold. **(D)** Shows the mean stimulus object position during the continuous phase for all participants, as well as the ± 3 and ± 10 std lines. The difficulty for the continuous phase is set to 30% of the user’s motor stability threshold (MST).

During calibration, the gain parameter was linearly increased over time so that even small corrections by the participant resulted in large changes in the stimulus position – thus systematically increasing difficulty. The gain of the system was characterized by a lambda (λ) parameter (Jex et al., 1966). If the participant failed to maintain the stimulus within a predefined spatial boundary (80% of distance from the center, or ± 22.28 DVA from center), it resulted in a “crash” and the circular stimulus was reset to the center of the screen. The position of the central stimulus and the user’s tracking position were continuously recorded at a sampling rate of 30 Hz. See Figure 1 for a schematic and equation describing the closed-loop unstable system that provides the dynamic conditions under which the participant must continuously provide corrective adjustments to stabilize the stimulus. See also Figure 2A for the cpCST task screen schematic.

The Continuous Performance Critical Stability Task (cpCST) employed a hybrid approach consisting of two distinct phases: an initial calibration phase that estimated the participant’s motor stability threshold, which was followed by a continuous performance phase in which they performed the task at a fixed difficulty level.

##### 2.3.1.1 Calibration phase

The calibration phase was similar to the original Jex et al. (1966) approach. Specifically, we employed a maximal performance to failure protocol similar to working memory tasks like digit span, and Corsi blocks (Corsi, 1972; Milner, 1971), and conceptually similar to the testing-the-limits approach (Kliegl et al., 1986).

During the calibration phase, participants attempted to maintain the central position of the stimulus by adaptively tilting the accelerometer device. Task difficulty (lambda) was linearly increased over time until the participant failed to control the stimulus - defined by the stimulus exceeding 80% of the distance from the center of the screen (crashed). See Figure 2B.

**FIGURE 3:**
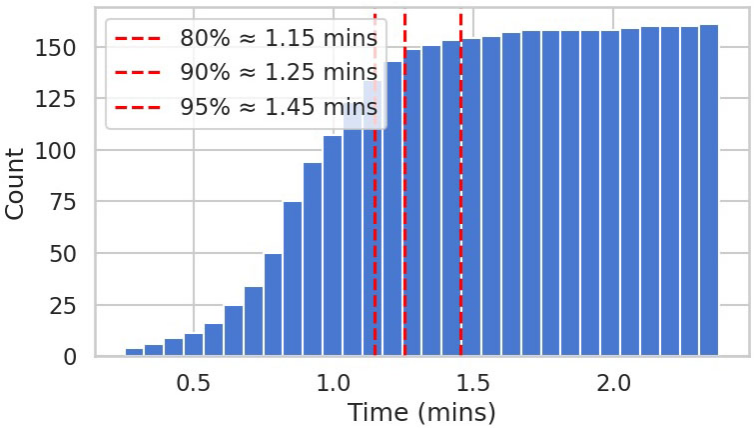
Cumulative count of participants (*N* = 166) who reached asymptotic performance on the calibration phase as a function of time in minutes. Vertical red lines indicate the time at which 80, 90, and 95% of participants reached asymptote, respectively.

Following failure, the stimulus was reset to the center of the screen, and the lambda parameter was reset to 50% of the value achieved at the time of the crash - allowing participants to “reset” and build back up to a higher difficulty. This process was repeated 10 times. We estimated each participant’s overall motor stability threshold (MST) by calculating the average lambda values reached over the final three calibration trials. Given the fixed number of calibration trials, we assessed whether the calibration phase was effective in reaching a stable estimate of each participant’s MST by calculating the amount of time required for each participant to reach asymptotic performance. Over 95% of participants reached asymptote within 1.5 min. Only two participants failed to reach asymptote by the final trial. We did not remove participants from continuous performance analyses based on this calibration metric. See Figure 3.

##### 2.3.1.2 Continuous performance phase

In the continuous performance phase, participants performed the same task as in the calibration phase. However, in this phase the difficulty level was held to just 30% of the participant’s individually estimated MST, and the trial duration was fixed at 10 min. See Figure 2C. To evaluate task compliance, we examined the participants’ mean position. Participants were able to maintain the position of the stimulus near the center of the screen, with an average distance of −0.156 ± 0.168 DVA across all participants. See Figure 2D.

#### 2.3.2 Flanker task

The flanker task was administered in a dedicated testing room in the CBIN at NKI. Participants performed a modified version of a flanker paradigm (Botvinick et al., 1999; Colcombe et al., 2004) in which they were asked to respond to a central target flanked by an array of distractors. Each trial presented one of three trial types: congruent, where the flanking stimuli matched the central target (e.g.,<<<<<); incongruent, where the flankers opposed the central target (e.g., <<><<); or neutral, where the flankers provided no directional information (e.g., - -<- -). Trial types were presented in equal proportions and were first-order counterbalanced to control for sequential effects.

The task consisted of a practice block of 30 trials with feedback. Each trial began with a fixation cross displayed for 500 ms, followed by the target stimulus. The inter-trial interval (ITI) averaged 1.16 s; mean total task duration was 618 s. Participants responded using a standard keyboard, pressing the “C” or “M” keys to indicate left or right central arrow directions, respectively. They were instructed to respond as quickly and accurately as possible. Participants were required to achieve at least 80% accuracy in the practice block to move on to the test phase. The test phase consisted of three blocks of 120 trials each, for a total of 360 trials. Participants were required to achieve at least 80% accuracy across all test trials to be included in analyses. Nine participants did not meet this minimum criteria.

#### 2.3.3 Woodcock Johnson Tests of Cognitive Abilities and Tests of Achievement (WJ)

Participants were administered a subset of the Woodcock Johnson Tests of Cognitive Abilities and Tests of Achievement (Schrank and Wendling, 2018) during in-person testing in a clinical research office conducted by research staff under the supervision of the study neuropsychologist. Tests were administered according to the standardized guidelines and data were entered into the publisher’s scoring program to generate composite scores used in this analysis. Brief Intellectual Ability (BIA) is an age-normalized composite score derived from the Oral Vocabulary, Number Series, and Verbal Attention subtests. Brief Achievement (ACHBRF) is an age-normalized composite score derived from the Letter-Word Identification, Applied Problems, and Spelling subtests. Published reliability for the BIA and ACHBRF are.92 to.95 and.96 to.97, respectively, across our analysis age range (Mcgrew et al., 2014).

#### 2.3.4 Cardiorespiratory fitness assessment (VO2max)

VO2max was estimated using the Parvo Medics True One 2400 Metabolic Measurement System (Crouter et al., 2006) which controlled a recumbent cycle ergometer in a dedicated physiological assessment laboratory at NKI. Participants exercised at a linearly increasing workload while their heart rate, exhaled CO_2_, and O_2_ were analyzed. The assessment was terminated when users met ≥ 90% of their age-related heart rate maximum (220-age) and a respiratory exchange ratio (CO2:O2 ratio; RER) ≥ 1.02, or voluntarily terminated the session.

### 2.4 Data analyses

#### 2.4.1 cpCST metrics

##### 2.4.1.1 Preprocessing

Raw stimulus coordinate data were preprocessed to correct for deviations caused by a crash during the continuous phase (*n* = 13 crashes). Crashes, identified as stimulus eccentricity exceeding ± 80% of the distance from the center to the edge of the screen were removed, and the removed data were reconstructed using piecewise polynomial interpolation (PCHIP) to ensure smooth continuity. Participants with two or more crashes were classified as outliers and removed (*n* = 5).

##### 2.4.1.2 Instantaneous reaction time (iRT)

**FIGURE 4:**
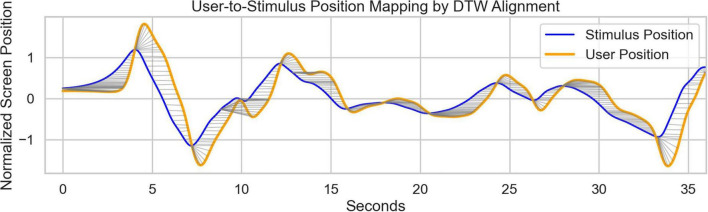
Illustration of the dynamic time warp (DTW) approach used to calculate the instantaneous reaction time (iRT) metric. The Z-scored positional time series for the stimulus position (blue) and the user position (orange) during the first 36 s of a participant’s Continuous Performance Critical Stability Task (cpCST) task performance are plotted above. User position time series was mirrored to align with the direction of the stimulus position time series. DTW was then applied to find the best fit transform between the user and stimulus position. The pointwise mapping of this transform between user and stimulus position are shown as light gray lines connecting corresponding points on the blue and orange lines. iRT at each timepoint is represented by the distance in time (x axis) between the corresponding points on each line.

To quantify temporal responsiveness during task performance, we computed an instantaneous reaction time (iRT) measure using dynamic time warping (DTW). This approach captured continuous time-varying latencies between stimulus and response movements by analyzing the x-coordinate (time) position vectors of both the stimulus object and user positions. The DTW algorithm identified the optimal alignment between these time series, producing a warp path representing temporal correspondence (See Figure 4). By multiplying x-coordinate distances by the sampling rate, we derived latency estimates for each timepoint, providing a highly granular measure of response latency. iRT computations were performed using custom Julia scripts and the DynamicAxisWarping.jl package (Carlson, 2020).

We then computed the mean iRT for each participant, and forwarded these to subsequent analyses.

#### 2.4.2 Flanker metrics

Accuracy and reaction time was recorded for each trial. Participants with accuracy below 80% across all trial conditions were classified as outliers and removed (*n* = 15). For each participant, incorrect responses were removed from further analysis. For correct trials, anticipatory RTs, defined as RTs faster than 200 ms, as well as RTs more than 2.5 SD longer than the participant’s mean were also removed from further analysis.

We computed the following metrics: mean reaction time for congruent (conRT) and incongruent (incRT) trials, and the standard flanker congruency effect (I-C; incongruent RT - congruent RT). These values were then forwarded for additional analysis.

#### 2.4.3 Reliability in cpCST and flanker

We computed split-half reliability estimates for both the cpCST iRT and the flanker task response times (conRT, incRT, and I-C). To estimate split-half reliability and generate population-level confidence intervals, we used a bootstrap procedure (Efron, 1992). In each bootstrap iteration, participants were sampled with replacement, and split-half reliability was computed using the permutation procedure, below.

Split-half reliability in each iteration, trials were randomly permuted and split into two halves. The aggregated mean was computed for each half, and the Pearson correlation between half-scores was calculated. The Spearman–Brown prophecy formula (Brown, 1910; Spearman, 1910) was applied to correct the correlation, providing an estimate of full-test reliability. This process was repeated 1,000 times, and the average split-half reliability was reported. The split-half approach provides an index of internal consistency by estimating how well two randomly chosen halves of the test relate to each other, scaled to reflect full-test reliability.

The resulting distribution of bootstrap estimates was used to derive 95% confidence intervals (2.5th and 97.5th percentiles).

All reliability estimates were computed using custom Python code, with bootstrap iterations parallelized using Joblib for computational efficiency. Random seeds were fixed to ensure reproducibility.

#### 2.4.4 Temporal efficiency in cpCST and flanker

##### 2.4.4.1 Stability curves

To evaluate the temporal efficiency of each task metric, we assessed how well early portions of the task captured participants’ overall response time (RT) profiles. For each participant, we computed the mean RT separately for each task and condition using only the first n minutes of task data (e.g., first 1, 2, 3, … 9 min). We then correlated these truncated means with the corresponding means computed using the full duration of the corresponding task. This yielded a curve of similarity (Pearson’s r) as a function of data collection time, providing an estimate of how quickly stable RT estimates emerge for cpCST iRT and flanker-based RT metrics.

##### 2.4.4.2 Comparison of stability curves

Statistical comparison between task stability curves for cpCST and flanker trial types was performed using Steiger’s Z-test for dependent correlations with one variable in common (Steiger, 1980). For each time point (1, 2, 3,. 9 min), we compared the correlation between the truncated and full dataset for the cpCST iRT against the corresponding correlation for each flanker task condition. This approach appropriately accounts for the repeated measures nature of the comparison, estimating the covariance between correlations and compensating for the correlation between the truncated measures (cpCST and flanker). This provides a more conservative and accurate assessment than treating the correlations as independent (Steiger, 1980). A significant Z-statistic indicates that one task achieves temporal stability more efficiently than the other at that specific time point.

#### 2.4.5 Predictive validity

To evaluate the predictive validity of the cpCST’s instantaneous reaction time (iRT), we conducted a series of regression analyses. Specifically, we examined whether the participants’ iRT could predict performance on proximal experimental measures of inhibitory control and attention (flanker task outcomes), distal clinical measures of cognitive performance (Woodcock-Johnson Cognition and Achievement composite scores), and a measure of central nervous system health and plasticity (VO2max). For each outcome variable, separate regression models were fitted using the mean iRT from the cpCST. We further explored the role of age, repeating these regression analyses both with and without age as a covariate in the models.

## 3 Results

Participants (N = 166) ranged in age from 18 to 76 years (M = 51.61, SD = 16.36) and reported 12 to 20 years of formal education (M = 15.81, SD = 2.11). The sample was 66% female (*n* = 110) and 34% male (*n* = 56). In terms of race, 81% identified as White (*n* = 134), 10% as Black or African American (*n* = 16), 5% as Asian (*n* = 8), 2% as American Indian or Alaska Native (*n* = 3), and 3% as multiracial (*n* = 5). Regarding ethnicity, 86% were Not Hispanic or Latino (*n* = 143), 13% were Hispanic or Latino (*n* = 22), and 0.6% preferred not to answer (*n* = 1).

### 3.1 Reliability of cpCST iRT and flanker outcomes

For the cpCST, the bootstrap-based estimate of population split-half reliability was high [r = 0.9993; 95% CI (0.999, 1.0)]. Split-half reliabilities were also strong for flanker conRT [0.9846; 95% CI: (0.9824–0.9868)] and incRT [r = 0.9752; 95% CI: (0.9725–0.9780)]. Although the split-half reliability for cpCST iRT was statistically greater than the flanker conRT and incRT (*p* < 0.05), the absolute difference (e.g., 0.9993 vs. 0.9842) is not likely meaningful.

We also assessed the reliability of the standard flanker congruency effect (I-C). The bootstrap-based reliability estimate for this difference score was significantly lower than the cpCST iRT or flanker conRT and incRTs [r = 0.8596; 95% CI: (0.8389–0.8805)].

### 3.2 Age and sex differences in cpCST and flanker measures

**FIGURE 5:**
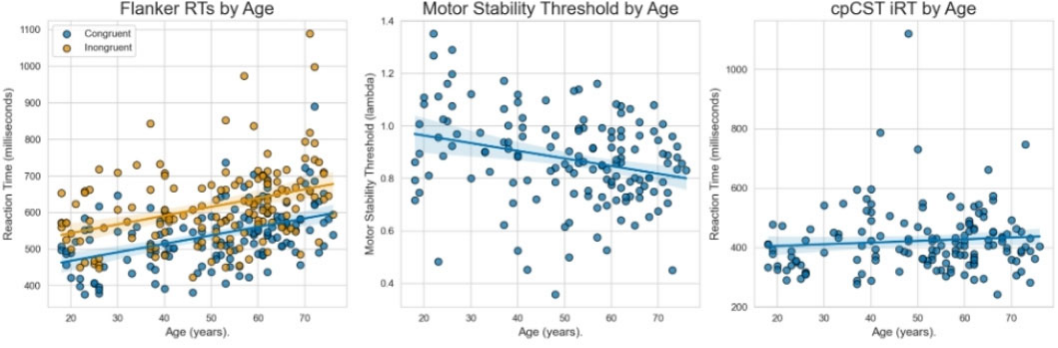
The association between age and the flanker task’s Congruent and Incongruent RTs, the cpCST’s Motor Stability Threshold, and cpCST iRT. Both flanker RTs and MST were significantly associated with age. The mean iRT was not significantly associated with age, suggesting that the calibration based on MST was successful.

To examine potential individual differences in the primary outcome measures, we performed a series of multiple regression analyses examining the impact of age on the cpCST and flanker measures. See Figure 5 for scatterplots of flanker RTs, cpCST motor stability threshold (MST) and instantaneous reaction time (iRT) as a function of age.

#### 3.2.1 cpCST measures

Age significantly predicted the Motor Stability Threshold [MST; B = −0.0025, *p* < 0.001; F(3, 142) = 57.17, *p* < 0.001, R^2^ = 0.55]. For instantaneous reaction time (iRT), age was not a significant predictor [B = 0.0006, *p* = 0.257; F(2, 143) = 3.36, *p* = 0.038, R^2^ = 0.05]. These results indicate that the calibration procedure effectively adjusted for well-documented age-related slowing throughout adulthood.

#### 3.2.2 Flanker measures

Age significantly predicted conRT [B = 2.35, *p* < 0.001; F(2, 143) = 19.99, *p* < 0.001, R^2^ = 0.22] and incRT [B = 2.43, *p* < 0.001; F(2, 143) = 11.94, *p* < 0.001, R^2^ = 0.14]. However, age was not a significant predictor of the I-C congruency effect [B = 0.08, *p* = 0.755; F(2, 143) = 0.07, *p* = 0.933, R^2^ = 0.001].

### 3.3 cpCST iRT predictive validity

To examine the relationship between cpCST iRT and each of our predicted metrics (Flanker, WJ, and VO2max), we conducted a series of linear regression analyses, both with and without age as a covariate.

#### 3.3.1 Flanker features

Continuous Performance Critical Stability Task iRT significantly predicted conRT [B = 112.48, *p* = 0.049; F(2, 143) = 20.96, *p* < 0.001, R^2^ = 0.23] and incRT [B = 171.91, *p* = 0.024; F(2, 143) = 14.02, *p* < 0.001, R^2^ = 0.16]. However, iRT did not significantly predict the I-C congruency effect [B = 59.42, *p* = 0.112; F(2, 143) = 1.33, *p* = 0.268, R^2^ = 0.02]. When age was included in the models, iRT continued to significantly predict conRT and incRT, while still failing to predict the I-C congruency effect.

Combined, these findings suggest that the cpCST iRT is more closely associated with the response generation aspects of flanker task performance rather than the inhibition of conflicting responses.

#### 3.3.2 WJ brief intellectual ability and WJ brief achievement

Instantaneous reaction time significantly predicted WJ Brief Intellectual Ability [BIA; B = −1.79, *p* = 0.004; F(2, 143) = 8.98, *p* < 0.001, R^2^ = 0.11] and WJ Brief Achievement [ACH; B = −1.42, *p* = 0.011; F(2, 134) = 8.74, *p* < 0.001, R^2^ = 0.12]. Faster iRT was associated with higher ability and achievement scores. Including age in the models did not eliminate these associations, suggesting that the relationships between iRT and the WJ outcome measures were not driven by age.

#### 3.3.3 VO2max

Mean iRT significantly predicted VO2max [B = −9.94, *p* = 0.010; F(2, 143) = 34.51, *p* < 0.001, R^2^ = 0.33]. When controlling for age, iRT remained a significant predictor of VO2max, demonstrating an association of faster reaction time speed with better aerobic capacity, beyond age-related effects.

### 3.4 Temporal efficiency

**FIGURE 6:**
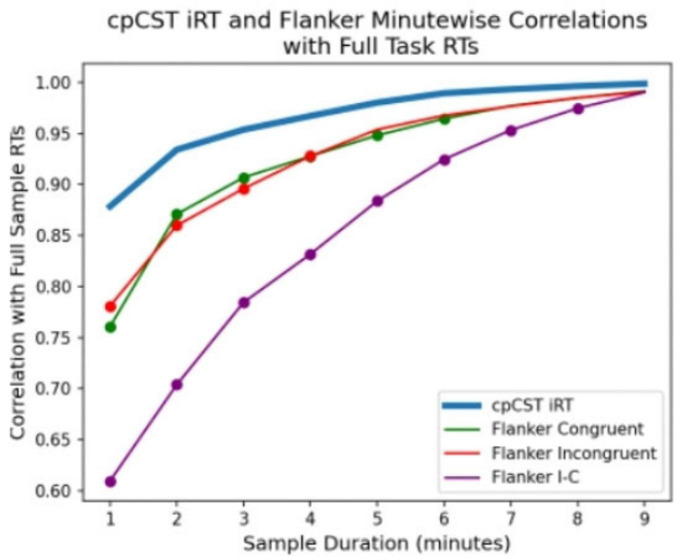
Correlation of Continuous Performance Critical Stability Task (cpCST) and flanker task mean reaction times at each minute of the task compared to full task performance. Note that cpCST iRT correlation (blue line) is highest over all durations of the task, while the standard flanker congruency effect (I–C; purple line) is lowest.

The statistical comparison of task stability curves described how well early segments of the task captured participants’ full-task response time (RT) characterizations. The correlation for each mean cumulative (1–9) minute segment of each task’s RT features are plotted below in Figure 6. Even 1 min of iRT data shows very good correlation with the full 10 min assessment (r = 0.87), and by the second minute the correlation with the full sample reached r = 0.94. The flanker Congruent and Incongruent RTs also performed well, though somewhat less well than the iRT. The I-C congruency contrast performed less well than either the iRT or the base flanker features. Locations denoted by a dot on each line show where the correlations for the flanker-based RT features are significantly lower than iRT, using Steiger’s Z-test for dependent correlations with one variable in common (Steiger, 1980).

## 4 Discussion

We introduced the Continuous Performance Critical Stability Task, offering high temporal precision of continuous psychomotor control across the lifespan. This report provides preliminary evidence for the reliability, predictive validity, and temporally efficiency of the cpCST – a potentially valuable complement to existing attention assessment paradigms. Below, we summarize key methodological innovations and psychometric properties, followed by implications for future research and clinical applications.

### 4.1 Methodological innovations

The cpCST incorporates three central methodological innovations.

High-density behavioral sampling (30 Hz) captures behavior at a granularity not possible with traditional discrete-response continuous performance tasks, which as noted above, typically sample at rates of 0.1–1 Hz [every 1–10 s; cf (Basner and Dinges, 2011; Conners et al., 2003; Dinges and Powell, 1985; Homack and Riccio, 2006)]. The enhanced temporal resolution provides data ideally suited to integrate with other data modalities such as EEG and physiological metrics - allowing sophisticated analyses of attentional stability and variability.

We also created a novel instantaneous reaction time measure, which estimates the temporal lag between the movement of a central stimulus object and the participant’s response to adjust to that movement. This approach estimates response time with high precision, reliability, and excellent temporal efficiency.

Additionally, the cpCST utilizes a hybrid design that combines adaptive calibration and subsequent fixed-difficulty assessments. Integrating the strengths of adaptive and fixed-difficulty paradigms provides individualized task difficulty while avoiding issues common in fully adaptive methods, such as oscillatory artifacts or instability (García-Pérez, 2011; Kontsevich and Tyler, 1999). It may also obviate the need for alternative task forms across groups with disparate baseline functioning, or in highly heterogeneous samples such as in aging, developmental, or lifespan studies.

### 4.2 Psychometric properties

The cpCST yielded high reliability estimates, with bootstrap-based split-half reliability greater than 0.999. High-density sampling and individualized calibration likely contributed to this reduced measurement error, facilitating the rapid detection of subtle individual differences (r > 0.9 after 1 min of data). This reliability may be especially advantageous in longitudinal studies or in interventions examining modest performance changes.

Age invariance is a notable strength of the cpCST. Although motor stability thresholds (MST) and traditional reaction time measures from the flanker task exhibited expected age-related slowing, cpCST’s iRT was stable across age. By calibrating task difficulty to each individual’s sensorimotor capacity, the cpCST appeared to effectively isolate attentional control from baseline sensorimotor function. This makes the task especially suitable in lifespan cognitive assessments, circumventing the need for distinct age-specific task versions.

The cpCST also exhibited robust validity across multiple domains. Significant associations with flanker conRT and incRT suggest convergent validity with aspects of attentional control. However, the lack of association with the flanker congruency effect may indicate that the cpCST primarily captures tonic aspects of attention (e.g., vigilance, sustained focus) rather than the application of inhibitory control processes. Head-to-head comparisons of cpCST performance metrics with established measures of vigiliance, sustained attention, and other dimensions of attentional control, while outside the scope of this analysis, are nevertheless warranted to characterize cpCST construct validity.

The temporal efficiency of the cpCST was also notable. Over 95% of participants reached asymptotic performance within the first 1.5 min of the calibration phase. Within 2 min of the continuous phase, the cpCST iRT exceeded an r = 0.9 correlation with full task performance. By comparison, the flanker trial types needed roughly 5 min of data to reach this level of association with the full flanker sample. This suggests the potential for cpCST to reduce task administration time without significant loss of information.

### 4.3 Implications and future directions

We identified significant predictive relationships across a broad range of domains, encompassing individual differences in low-level physiological functioning (VO2max), reaction time in a traditional cognitive task (flanker), and even global estimates of intellectual ability and achievement (WJ Brief Intellectual Ability, Brief Achievement). While speculative, this remarkable range of associations suggests that the cpCST may tap one or more central aspects of neurocognitive functioning. Future research to better contextualize the cpCST amongst the existing constellation of cognitive assessments will likely be of high value.

The central features of the cpCST position it as a promising tool for research and clinical settings. Its high temporal resolution enables tighter integration with physiological measures (e.g., EEG, fMRI, heart rate, skin conductance), facilitating exploration of neural mechanisms underlying moment-to-moment attention variability, and “brain-body” interactions. Additionally, its individualized calibration method is likely to prove valuable in heterogeneous clinical populations or lifespan studies, as it reduces confounds related to sensorimotor speed differences or ceiling/floor effects.

**FIGURE 7:**
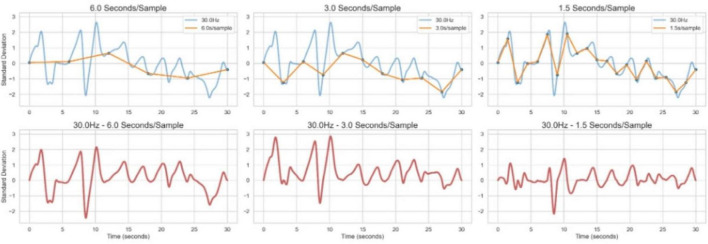
Illustration of how dense sampling of behavior may more efficiently characterize an individual’s attentional functioning. The top row shows the Z-scaled spatial path of a participant’s stimulus object (SO) over time (blue line). The SO drifts away from center (zero on the Y axis) and is subsequently returned to center; behavior is sampled at 30 Hz and demonstrates a rich pattern of change over time. The top row also shows that trajectory of behavior, but sampled at rates in the range of standard discrete reaction time (RT) paradigms such as the PVT and Conners CPT (orange lines; 6.0, 3, and 1.5 s, left to right), demonstrating a much simplified pattern of apparent behavior. In the bottom row, we subtract the simulated discrete RT behavior from the same behavior as sampled at 30 Hz in the Continuous Performance Critical Stability Task (cpCST). Examining these plots, it is evident that much of the behavioral variation evident in the cpCST remains undescribed if sampled in the typical discrete RT task sampling temporal regimes. This is most evident when sampled every 6 s. However, even when the sampling rate is increased to 1.5 s and the orange line more closely matches the 30 Hz blue line, the subtraction plotted in red reveals non-trivial variation. In all plots periods of a participant’s behavioral variation greater than two standard deviations would remain unaccounted under traditional discrete RT paradigms. This improved precision in assessment may help to explain the cpCST’s high split-half reliability and temporal efficiency.

The task’s temporal efficiency and straightforward administration suggest suitability for large-scale assessments, longitudinal monitoring, and remote or mobile implementations. Future studies should explicitly evaluate cpCST’s sensitivity to attentional changes resulting from interventions (e.g., sleep deprivation, stimulant medication, cognitive training) and establish its utility in diverse clinical populations (e.g., ADHD, TBI, MCI). Additionally, as illustrated in Figure 7, the high density sampling may allow detection of subtle behavioral dynamics not captured with discrete response paradigms - which may not only contribute to the cpCST’s relatively high temporal efficiency and reliability, but also allow for new insights into attentional dynamics.

Finally, the rich, high-density behavioral data generated by the cpCST is well-suited for computational modeling approaches, such as drift diffusion models or Bayesian frameworks. Future work could leverage these modeling techniques to better characterize the attentional process dynamics captured by the cpCST.

### 4.4 Limitations

Several limitations should be acknowledged. Although predictive validity and split-half reliability were established, the cpCST’s sensitivity to intervention-induced attentional changes remains to be validated. As noted in Psychometric Properties, above, cpCST task performance was not directly compared to a full range of established measures of sustained attention or attentional control. Future work that comprehensively reviews the theoretical positioning of widely adopted and emerging attention tasks and provides psychometric evaluation via head-to-head empirical evidence for both shared and unique behavioral features would provide useful information to guide research advances in theoretical and practical applications. Our current analysis age range (18–76 years) is substantial, but was undertaken as a preliminary convenience sample; larger samples that include evaluation of efficacy and validity in younger and older individuals require further examination. Likewise, this is a community-based normative sample and psychometric properties should be evaluated across different clinical populations. Given the cross-sectional nature of our sample, we can only establish internal reliability through bootstrap methods. Future work is needed to examine test-retest reliability under frameworks like the intraclass correlation coefficient [ICC; (Shrout and Fleiss, 1979; Zuo and Xing, 2014)].

Additionally, while we include summary evidence of calibration feasibility and sensitivity, a full psychometric evaluation of the calibration phase (e.g., MST distributions, convergence dynamics, and predictive validity) is beyond the scope of this initial paper and will be presented in a companion manuscript.

Finally, while high-density behavioral sampling offers analytical richness, the relative complexity of calculating iRT using dynamic time warping (DTW) may present obstacles to widespread adoption. To address this, we will provide streamlined and containerized analysis pipelines on GitHub. Developing accessible pipelines and normative databases will be essential for broader clinical adoption and research utilization.

## 5 Conclusion

The Continuous Performance Critical Stability Task introduces methodological advances in the assessment of attention. Its exceptional reliability, age invariance, predictive validity, and temporal efficiency address limitations in existing measures. Future validation efforts integrating physiological measures, computational modeling, and diverse clinical applications will further establish cpCST’s utility as an essential tool for attention research and assessment.

## Data Availability

The datasets presented in this study can be found in online repositories. The names of the repository/repositories and accession number(s) can be found below: the datasets generated for this study can be found in the NKI-Rockland Sample data resource https://rocklandsample.org/. Access to raw data requires completion of a data use agreement. Analysis datasets used for the current study are available from the corresponding author upon reasonable request.

## References

[B1] Anderson B. A. (2021). An adaptive view of attentional control. *Am. Psychol.* 76 1410–1422. 10.1037/amp0000917 35266739 PMC9295553

[B2] Attarha M. Mahncke H. Merzenich M. (2024). The real-world usability, feasibility, and performance distributions of deploying a digital toolbox of computerized assessments to remotely evaluate brain health: Development and usability study. *JMIR Format. Res.* 8:e53623. 10.2196/53623 38739916 PMC11130778

[B3] Baddeley A. (1996). Exploring the central executive. *Q. J. Exp. Psychol. Hum. Exp. Psychol.* 49 5–28. 10.1080/713755608

[B4] Barbey F. M. Farina F. R. Buick A. R. Danyeli L. Dyer J. F. Islam M. N. (2022). Neuroscience from the comfort of your home: Repeated, self-administered wireless dry EEG measures brain function with high fidelity. *Front. Digit. Health* 4:944753. 10.3389/fdgth.2022.944753 35966140 PMC9372279

[B5] Barriga A. Q. Doran J. W. Newell S. B. Morrison E. M. Barbetti V. Dean Robbins B. (2002). Relationships between problem behaviors and academic achievement in adolescents: The unique role of attention problems. *J. Emot. Behav. Disord.* 10 233–240. 10.1177/10634266020100040501

[B6] Basner M. Dinges D. F. (2011). Maximizing sensitivity of the psychomotor vigilance test (PVT) to sleep loss. *Sleep* 34 581–591. 10.1093/sleep/34.5.581 21532951 PMC3079937

[B7] Beck L. H. Bransome E. D. Jr. Mirsky A. F. Rosvold H. E. Sarason I. (1956). A continuous performance test of brain damage. *J. Consult. Psychol.* 20 343–350. 10.1037/h0043220 13367264

[B8] Best J. R. Miller P. H. (2010). A developmental perspective on executive function: Development of executive functions. *Child Dev.* 81 1641–1660. 10.1111/j.1467-8624.2010.01499.x 21077853 PMC3058827

[B9] Bogdanova Y. Yee M. K. Ho V. T. Cicerone K. D. (2016). Computerized cognitive rehabilitation of attention and executive function in acquired brain injury: A systematic review. *J. Head Trauma Rehabil.* 31 419–433. 10.1097/HTR.0000000000000203 26709580 PMC5401713

[B10] Botvinick M. Nystrom L. E. Fissell K. Carter C. S. Cohen J. D. (1999). Conflict monitoring versus selection-for-action in anterior cingulate cortex. *Nature* 402 179–181. 10.1038/46035 10647008

[B11] Brown W. (1910). Some experimental results in the correlation of mental abilities. *Br. J. Psychol.* 3 296–322. 10.1111/j.2044-8295.1910.tb00207.x

[B12] Carlson F. B. (2020). *DynamicAxisWarping.jl.* Available online at: https://github.com/baggepinnen/DynamicAxisWarping.jl

[B13] Castellanos F. X. Sonuga-Barke E. J. S. Scheres A. Di Martino A. Hyde C. Walters J. R. (2005). Varieties of attention-deficit/hyperactivity disorder-related intra-individual variability. *Biol. Psychiatry* 57 1416–1423. 10.1016/j.biopsych.2004.12.005 15950016 PMC1236991

[B14] Cerella J. (1990). “Aging and Information-Processing Rate,” in *Handbook of the psychology of aging*, eds Birren J. E. Schaie K. W. (Amsterdam: Elsevier), 201–221. 10.1016/b978-0-12-101280-9.50018-8

[B15] Chen A. Wang A. Wang T. Tang X. Zhang M. (2017). Behavioral oscillations in visual attention modulated by task difficulty. *Front. Psychol.* 8:1630. 10.3389/fpsyg.2017.01630 29018373 PMC5622979

[B16] Colcombe S. J. Erickson K. I. Scalf P. E. Kim J. S. Prakash R. McAuley E. (2006). Aerobic exercise training increases brain volume in aging humans. *J. Gerontol. A Biol. Sci. Med. Sci.* 61 1166–1170. 10.1093/gerona/61.11.1166 17167157

[B17] Colcombe S. J. Kramer A. F. Erickson K. I. Scalf P. McAuley E. Cohen N. J. (2004). Cardiovascular fitness, cortical plasticity, and aging. *Proc. Natl. Acad. Sci. U.S.A.* 101 3316–3321. 10.1073/pnas.0400266101 14978288 PMC373255

[B18] Colcombe S. Kramer A. (2003). Fitness effects on the cognitive function of older adults a meta-analytic study. *Psychol. Sci.* 14 125–130. 10.1111/1467-9280.t01-1-01430 12661673

[B19] Colino F. L. Howse H. Norton A. Trska R. Pluta A. Luehr S. J. C. (2017). Older adults display diminished error processing and response in a continuous tracking task. *Psychophysiology* 54 1706–1713. 10.1111/psyp.12907 28621460

[B20] Conners C. K. Epstein J. N. Angold A. Klaric J. (2003). Continuous performance test performance in a normative epidemiological sample. *J. Abnorm. Child Psychol.* 31 555–562. 10.1023/a:1025457300409 14561062

[B21] Conway A. R. A. Kane M. J. Engle R. W. (2003). Working memory capacity and its relation to general intelligence. *Trends Cogn. Sci.* 7 547–552. 10.1016/j.tics.2003.10.005 14643371

[B22] Cooper S. R. Gonthier C. Barch D. M. Braver T. S. (2017). The role of psychometrics in individual differences research in cognition: A case study of the AX-CPT. *Front. Psychol.* 8:1482. 10.3389/fpsyg.2017.01482 28928690 PMC5591582

[B23] Corsi P. M. (1972). *Human memory and the medial temporal region of the brain.* Montreal, QC: McGill University, 34.

[B24] Cowan N. Morey C. C. AuBuchon A. M. Zwilling C. E. Gilchrist A. L. (2010). Seven-year-olds allocate attention like adults unless working memory is overloaded: Capacity and attention allocation. *Dev. Sci.* 13 120–133. 10.1111/j.1467-7687.2009.00864.x 20121868 PMC2819460

[B25] Craik F. I. M. (1986). “A functional account of age differences in memory,” in *Human memory and cognitive capabilities, mechanisms and performances*, eds Klix F. Hagendorf H. (Amsterdam: Elsevier), 409–422.

[B26] Crouter S. E. Antczak A. Hudak J. R. DellaValle D. M. Haas J. D. (2006). Accuracy and reliability of the ParvoMedics TrueOne 2400 and MedGraphics VO2000 metabolic systems. *Eur. J. Appl. Physiol.* 98 139–151. 10.1007/s00421-006-0255-0 16896734

[B27] Davis E. T. Fujawa G. Shikano T. (2002). Perceptual processing and search efficiency of young and older adults in a simple-feature search task: A staircase approach. *J. Gerontol. Ser. B Psychol. Sci. Soc. Sci.* 57 324–337. 10.1093/geronb/57.4.p324 12084783

[B28] de Souza Almeida R. Faria A. Klein R. M. (2021). On the origins and evolution of the Attention Network Tests. *Neurosci. Biobehav. Rev.* 126 560–572. 10.1016/j.neubiorev.2021.02.028 33766674

[B29] Decker A. Dubois M. Duncan K. Finn A. S. (2023). Pay attention and you might miss it: Greater learning during attentional lapses. *Psychon. Bull. Rev.* 30 1041–1052. 10.3758/s13423-022-02226-6 36510094

[B30] Denckla M. B. (1996). Biological correlates of learning and attention: What is relevant to learning disability and attention-deficit hyperactivity disorder? *J. Dev. Behav. Pediatr.* 17:114. 10.1097/00004703-199604000-00011 8727849

[B31] Di Martino A. Ghaffari M. Curchack J. Reiss P. Hyde C. Vannucci M. (2008). Decomposing intra-subject variability in children with attention-deficit/hyperactivity disorder. *Biol. Psychiatry* 64 607–614. 10.1016/j.biopsych.2008.03.008 18423424 PMC2707839

[B32] DiFrancesco M. W. Van Dyk T. Altaye M. Drummond S. P. A. Beebe D. W. (2019). Network-based responses to the psychomotor vigilance task during lapses in adolescents after short and extended sleep. *Sci. Rep.* 9:13913. 10.1038/s41598-019-50180-6 31558730 PMC6763427

[B33] Dinges D. F. Powell J. W. (1985). Microcomputer analyses of performance on a portable, simple visual RT task during sustained operations. *Behav. Res. Methods nstruments Comput.* 17 652–655. 10.3758/bf03200977

[B34] Draheim C. Tshukara J. S. Engle R. W. (2024). Replication and extension of the toolbox approach to measuring attention control. *Behav. Res. Methods* 56 2135–2157. 10.3758/s13428-023-02140-2 37253957 PMC10228888

[B35] Draheim C. Tsukahara J. S. Martin J. D. Mashburn C. A. Engle R. W. (2021). A toolbox approach to improving the measurement of attention control. *J. Exp. Psychol. Gen.* 150 242–275. 10.1037/xge0000783 32700925

[B36] Duncan J. (1986). Disorganization of behavior after frontal lobe damage. *Cogn. Neuropsychol.* 3 271–290. 10.1080/02643298608253360

[B37] Efron B. (1992). “Bootstrap methods: Another look at the jackknife,” in *Springer Series in Statistics* (New York, NY: Springer), 569–593. 10.1007/978-1-4612-4380-9_41

[B38] Esterman M. Noonan S. K. Rosenberg M. Degutis J. (2013). In the zone or zoning out? Tracking behavioral and neural fluctuations during sustained attention. *Cereb. Cortex* 23 2712–2723. 10.1093/cercor/bhs261 22941724

[B39] Ewolds H. E. Bröker L. de Oliveira R. F. Raab M. Künzell S. (2017). Implicit and explicit knowledge both improve dual task performance in a continuous pursuit tracking task. *Front. Psychol.* 8:2241. 10.3389/fpsyg.2017.02241 29312083 PMC5744266

[B40] Fair D. A. Bathula D. Nikolas M. A. Nigg J. T. (2012). Distinct neuropsychological subgroups in typically developing youth inform heterogeneity in children with ADHD. *Proc. Natl. Acad. Sci. U.S.A.* 109 6769–6774. 10.1073/pnas.1115365109 22474392 PMC3340031

[B41] Farahbakhsh M. Dekker T. M. Jones P. R. (2019). Psychophysics with children: Evaluating the use of maximum likelihood estimators in children aged 4-15 years (QUEST+). *J. Vis.* 19:22. 10.1167/19.6.22 31246228

[B42] Fleming S. M. Weil R. S. Nagy Z. Dolan R. J. Rees G. (2010). Relating introspective accuracy to individual differences in brain structure. *Science* 329 1541–1543. 10.1126/science.1191883 20847276 PMC3173849

[B43] Fortenbaugh F. C. DeGutis J. Germine L. Wilmer J. B. Grosso M. Russo K. (2015). Sustained attention across the life span in a sample of 10,000: Dissociating ability and strategy: Dissociating ability and strategy. *Psychol. Sci.* 26 1497–1510. 10.1177/0956797615594896 26253551 PMC4567490

[B44] García-Pérez M. A. (2011). A cautionary note on the use of the adaptive up-down method. *J. Acoust. Soc. Am.* 130 2098–2107. 10.1121/1.3628334 21973364

[B45] Gershon R. C. Cella D. Fox N. A. Havlik R. J. Hendrie H. C. Wagster M. V. (2010). Assessment of neurological and behavioural function: The NIH toolbox. *Lancet Neurol.* 9 138–139. 10.1016/S1474-4422(09)70335-7 20129161

[B46] Gibbons R. D. Lauderdale D. S. Wilson R. S. Bennett D. A. Arar T. Gallo D. A. (2024). Adaptive measurement of cognitive function based on multidimensional item response theory. *Alzheimers Dement.* 10:e70018. 10.1002/trc2.70018 39748843 PMC11694520

[B47] Gronwall D. M. (1977). Paced auditory serial-addition task: A measure of recovery from concussion. *Percept. Mot. Skills* 44 367–373. 10.2466/pms.1977.44.2.367 866038

[B48] Gross J. J. (2015). Emotion regulation: Current status and future prospects. *Psychol. Inq.* 26 1–26. 10.1080/1047840X.2014.940781

[B49] Halperin J. M. (1991). The clinical assessment of attention. *Int. J. Neurosci.* 58 171–182. 10.3109/00207459108985433 1365040

[B50] Hedge C. Powell G. Sumner P. (2018). The reliability paradox: Why robust cognitive tasks do not produce reliable individual differences. *Behav. Res. Methods* 50 1166–1186. 10.3758/s13428-017-0935-1 28726177 PMC5990556

[B51] Homack S. Riccio C. A. (2006). Conners’ continuous performance test. *J. Attent. Disord.* 9 556–558. 10.1177/1087054705283578 16481673

[B52] Huang R.-S. Jung T.-P. Makeig S. (2005). “Analyzing event-related brain dynamics in continuous compensatory tracking tasks,” in *Proceedings of the annual international conference of the IEEE engineering in medicine and biology society*, (Shanghai), 5750–5753. 10.1109/IEMBS.2005.1615794 17281564

[B53] Jackson J. D. Balota D. A. (2012). Mind-wandering in younger and older adults: Converging evidence from the Sustained Attention to Response Task and reading for comprehension. *Psychol. Aging* 27 106–119. 10.1037/a0023933 21707183 PMC3508668

[B54] Jacobson L. A. Ryan M. Martin R. B. Ewen J. Mostofsky S. H. Denckla M. B. (2011). Working memory influences processing speed and reading fluency in ADHD. *Child Neuropsychol.* 17 209–224. 10.1080/09297049.2010.532204 21287422 PMC3309419

[B55] Jex H. R. McDonnell J. D. Phatak A. V. (1966). A “critical” tracking task for man-machine research related to the operator’s effective delay time. I. Theory and experiments with a first-order divergent controlled element. NASA CR-616. *NASA Contract Rep. NASA CR* 1–105. 5297174

[B56] Kail R. (1991). Processing time declines exponentially during childhood and adolescence. *Dev. Psychol.* 27 259–266. 10.1037/0012-1649.27.2.259

[B57] Kinsella G. J. (1998). Assessment of attention following traumatic brain injury: A review. *Neuropsychol. Rehabil.* 8 351–375. 10.1080/713755576

[B58] Klee S. H. Garfinkel B. D. (1983). The computerized continuous performance task: A new measure of inattention. *J. Abnorm. Child Psychol.* 11 487–495. 10.1007/bf00917077 6689172

[B59] Klein A. Clucas J. Krishnakumar A. Ghosh S. S. Van Auken W. Thonet B. (2020). Remote digital psychiatry for mobile mental health assessment and therapy: MindLogger platform development study. *J. Med. Internet Res.* 23:e22369. 10.2196/preprints.22369 PMC8663601 34762054

[B60] Kliegl R. Smith J. Baltes P. B. (1986). *Testing-the-limits, expertise, and memory in adulthood and old age.* Potsdam: Universität Potsdam.

[B61] Kontsevich L. L. Tyler C. W. (1999). Bayesian adaptive estimation of psychometric slope and threshold. *Vis. Res.* 39 2729–2737. 10.1016/s0042-6989(98)00285-5 10492833

[B62] Leark R. A. Greenberg L. Corman C. (1997). Development of six new scales for the test of variables of attention. *Arch. Clin. Neuropsychol.* 12 354–355. 10.1093/arclin/12.4.354

[B63] Lindenberger U. Baltes P. B. (1997). Intellectual functioning in old and very old age: Cross-sectional results from the Berlin aging study. *Psychol. Aging* 12 410–432. 10.1037/0882-7974.12.3.410 9308090

[B64] Lindfield K. C. Wingfield A. Bowles N. L. (1994). Identification of fragmented pictures under ascending versus fixed presentation in young and elderly adults: Evidence for the inhibition-deficit hypothesis. *Neuropsychol. Dev. Cogn.* 1 282–291. 10.1080/13825589408256582

[B65] Logan G. D. Cowan W. B. (1984). On the ability to inhibit thought and action: A theory of an act of control. *Psychol. Rev.* 91 295–327. 10.1037/0033-295x.91.3.295 24490789

[B66] Logan S. Baier M. P. Owen D. B. Peasari J. Jones K. L. Ranjit R. (2023). Cognitive heterogeneity reveals molecular signatures of age-related impairment. *PNAS Nexus* 2:gad101. 10.1093/pnasnexus/pgad101 37091543 PMC10118303

[B67] Lopez-Garcia P. Lesh T. A. Salo T. Barch D. M. MacDonald A. W. III Gold J. M. (2016). The neural circuitry supporting goal maintenance during cognitive control: A comparison of expectancy AX-CPT and dot probe expectancy paradigms. *Cogn. Affect. Behav. Neurosci.* 16 164–175. 10.3758/s13415-015-0384-1 26494483 PMC4819423

[B68] Mackworth N. H. (1948). The breakdown of vigilance during prolonged visual search. *Q. J. Exp. Psychol.* 1 6–21. 10.1080/17470214808416738

[B69] Manning C. Jones P. R. Dekker T. M. Pellicano E. (2018). Psychophysics with children: Investigating the effects of attentional lapses on threshold estimates. *Attent. Percept. Psychophys.* 80 1311–1324. 10.3758/s13414-018-1510-2 29582387 PMC6060997

[B70] Mcgrew K. S. Laforte E. M. Schrank F. A. (2014). *Technical manual: Woodcock-Johnson IV.*

[B71] Milner B. (1971). Interhemispheric differences in the localization of psychological processes in man. *Br. Med. Bull.* 27 272–277. 10.1093/oxfordjournals.bmb.a070866 4937273

[B72] Nooner K. B. Colcombe S. J. Tobe R. H. Mennes M. Benedict M. M. Moreno A. L. (2012). The NKI-rockland sample: A model for accelerating the pace of discovery science in psychiatry. *Front. Neurosci.* 6:152. 10.3389/fnins.2012.00152 23087608 PMC3472598

[B73] Norman D. A. Shallice T. (1986). “Attention to action: Willed and automatic control of behavior,” in *Consciousness and self-regulation*, eds Davidson R. J. Schwartz G. E. Shapiro D. (Boston, MA: Springer US), 1–18. 10.1007/978-1-4757-0629-1_1

[B74] Owsley C. (2016). Vision and aging. *Annu. Rev. Vis. Sci.* 2 255–271. 10.1146/annurev-vision-111815-114550 28532355

[B75] Parsons S. Kruijt A.-W. Fox E. (2019). Psychological science needs a standard practice of reporting the reliability of cognitive-behavioral measurements. *Adv. Methods Pract. Psychol. Sci.* 2 378–395. 10.1177/2515245919879695

[B76] Posner M. I. Petersen S. E. (1990). The attention system of the human brain. *Annu. Rev. Neurosci.* 13 25–42. 10.1146/annurev.ne.13.030190.000325 2183676

[B77] Prakash R. S. Snook E. M. Erickson K. I. Colcombe S. J. Voss M. W. Motl R. W. (2007). Cardiorespiratory fitness: A predictor of cortical plasticity in multiple sclerosis. *Neuroimage* 34 1238–1244. 10.1016/j.neuroimage.2006.10.003 17134916

[B78] Quick K. M. Mischel J. L. Loughlin P. J. Batista A. P. (2018). The critical stability task: Quantifying sensory-motor control during ongoing movement in nonhuman primates. *J. Neurophysiol.* 120 2164–2181. 10.1152/jn.00300.2017 29947593 PMC6295547

[B79] Racer K. H. Dishion T. J. (2012). Disordered attention: Implications for understanding and treating internalizing and externalizing disorders in childhood. *Cogn. Behav. Pract.* 19 31–40. 10.1016/j.cbpra.2010.06.005 23365493 PMC3556924

[B80] Ramaekers J. G. Kauert G. van Ruitenbeek P. Theunissen E. L. Schneider E. Moeller M. R. (2006). High-potency marijuana impairs executive function and inhibitory motor control. *Neuropsychopharmacology* 31 2296–2303. 10.1038/sj.npp.1301068 16572123

[B81] Reuter-Lorenz P. A. Cappell K. A. (2008). Neurocognitive aging and the compensation hypothesis. *Curr. Dir. Psychol. Sci.* 17 177–182. 10.1111/j.1467-8721.2008.00570.x

[B82] Robertson I. H. Manly T. Andrade J. Baddeley B. T. Yiend J. (1997). ‘Oops!’: Performance correlates of everyday attentional failures in traumatic brain injured and normal subjects. *Neuropsychologia* 35 747–758. 10.1016/s0028-3932(97)00015-8 9204482

[B83] Roheger M. Meyer J. Kessler J. Kalbe E. (2020). Predicting short- and long-term cognitive training success in healthy older adults: Who benefits? *Neuropsychol. Dev. Cogn.* 27 351–369. 10.1080/13825585.2019.1617396 31092117

[B84] Rosenberg M. Noonan S. DeGutis J. Esterman M. (2013). Sustaining visual attention in the face of distraction: A novel gradual-onset continuous performance task. *Attent. Percept. Psychophys.* 75 426–439. 10.3758/s13414-012-0413-x 23299180

[B85] Rueda M. R. Fan J. McCandliss B. D. Halparin J. D. Gruber D. B. Lercari L. P. (2004). Development of attentional networks in childhood. *Neuropsychologia* 42 1029–1040. 10.1016/j.neuropsychologia.2003.12.012 15093142

[B86] Sadeghi M. Sharif Razavian R. Bazzi S. Chowdhury R. H. Batista A. P. Loughlin P. J. (2024). Inferring control objectives in a virtual balancing task in humans and monkeys. *eLife* 12:R88514. 10.7554/eLife.88514 38738986 PMC11090506

[B87] Sahakian B. J. Owen A. M. (1992). Computerized assessment in neuropsychiatry using CANTAB: Discussion paper. *J. R. Soc. Med.* 85 399–402. 1629849 PMC1293547

[B88] Salthouse T. A. (1996). The processing-speed theory of adult age differences in cognition. *Psychol. Rev.* 103 403–428. 10.1037/0033-295x.103.3.403 8759042

[B89] Schmidt J. da Silva Senges G. Gonçalves Fernandes Campos R. Lucieri Alonso G. (2024). Sustained attention can be measured using a brief computerized attention task. *Sci. Rep.* 14:17001. 10.1038/s41598-024-68093-4 39043835 PMC11266567

[B90] Schneiders J. A. Opitz B. Krick C. M. Mecklinger A. (2011). Separating intra-modal and across-modal training effects in visual working memory: An fMRI investigation. *Cereb. Cortex* 21 2555–2564. 10.1093/cercor/bhr037 21471559

[B91] Schrank F. A. Wendling B. J. (2018). “The Woodcock–Johnson IV: Tests of cognitive abilities, tests of oral language, tests of achievement,” in *Contemporary intellectual assessment: Theories, tests, and issues 4th Edition*, eds Flanagan D. P. McDonough E. M. (The Guilford Press), 383–451. Available online at: https://psycnet.apa.org/record/2018-36604-014

[B92] Servan-Schreiber D. Cohen J. D. Steingard S. (1996). Schizophrenic deficits in the processing of context. A test of a theoretical model. *Arch. Gen. Psychiatry* 53 1105–1112. 10.1001/archpsyc.1996.01830120037008 8956676

[B93] Shi R. Sharpe L. Abbott M. (2019). A meta-analysis of the relationship between anxiety and attentional control. *Clin. Psychol. Rev.* 72:101754. 10.1016/j.cpr.2019.101754 31306935

[B94] Shrout P. E. Fleiss J. L. (1979). Intraclass correlations: Uses in assessing rater reliability. *Psychol. Bull.* 86 420–428. 10.1037/0033-2909.86.2.420 18839484

[B95] Smallwood J. Davies J. B. Heim D. Finnigan F. Sudberry M. O’Connor R. (2004). Subjective experience and the attentional lapse: Task engagement and disengagement during sustained attention. *Conscious. Cogn.* 13 657–690. 10.1016/j.concog.2004.06.003 15522626

[B96] Snow N. J. Mang C. S. Roig M. McDonnell M. N. Campbell K. L. Boyd L. A. (2016). The effect of an acute bout of moderate-intensity aerobic exercise on motor learning of a continuous tracking task. *PLoS One* 11:e0150039. 10.1371/journal.pone.0150039 26901664 PMC4764690

[B97] Sorrel M. A. Barrada J. R. de la Torre J. Abad F. J. (2020). Adapting cognitive diagnosis computerized adaptive testing item selection rules to traditional item response theory. *PLoS One* 15:e0227196. 10.1371/journal.pone.0227196 31923227 PMC6953845

[B98] Spearman C. (1910). Correlation calculated from faulty data. *Br. J. Psychol.* 3 271–295. 10.1111/j.2044-8295.1910.tb00206.x

[B99] Steiger J. H. (1980). Tests for comparing elements of a correlation matrix. *Psychological Bulletin* 87, 245–251. 10.1037/0033-2909.87.2.245

[B100] Stierwalt J. A. G. Murray L. L. (2002). Attention impairment following traumatic brain injury. *Semin. Speech Lang.* 23 129–138. 10.1055/s-2002-24989 11951173

[B101] Tipper S. P. Bourque T. A. Anderson S. H. Brehaut J. C. (1989). Mechanisms of attention: A developmental study. *J. Exp. Child Psychol.* 48 353–378. 10.1016/0022-0965(89)90047-7 2584921

[B102] Treutwein B. (1995). Adaptive psychophysical procedures. *Vis. Res.* 35 2503–2522. 10.1016/0042-6989(95)00016-s 8594817

[B103] Unsworth N. Miller A. L. Strayer D. L. (2024). Individual differences in attention control: A meta-analysis and re-analysis of latent variable studies. *Psychon. Bull. Rev.* 31 2487–2533. 10.3758/s13423-024-02516-1 38769271

[B104] Verhaeghen P. Cerella J. (2008). “Everything we know about aging and response times: A meta-analytic integration,” in *Handbook of cognitive aging: Interdisciplinary perspectives*, eds Hofer M. Alwin D. F. (New York, NY: SAGE Publications, Inc), 134–150. 10.4135/9781412976589.n8

[B105] von Bastian C. C. Blais C. Brewer G. A. Gyurkovics M. Hedge C. Kałamała P. (2020). Advancing the understanding of individual differences in attentional control: Theoretical, methodological, and analytical considerations. *PsyArXiv* [Preprint]. 10.31234/osf.io/x3b9k

[B106] Wingfield A. Tun P. A. McCoy S. L. (2005). Hearing loss in older adulthood: What it is and how it interacts with cognitive performance. *Curr. Dir. Psychol. Sci.* 14 144–148. 10.1111/j.0963-7214.2005.00356.x

[B107] Yang C. S. Cowan N. J. Haith A. M. (2021). De novo learning versus adaptation of continuous control in a manual tracking task. *eLife* 10:e62578. 10.7554/eLife.62578 34169838 PMC8266385

[B108] Yangüez M. Bediou B. Chanal J. Bavelier D. (2024). In search of better practice in executive functions assessment: Methodological issues and potential solutions. *Psychol. Rev.* 131 402–430. 10.1037/rev0000434 37616099

[B109] Zuo X.-N. Xing X.-X. (2014). Test-retest reliabilities of resting-state FMRI measurements in human brain functional connectomics: A systems neuroscience perspective. *Neurosci. Biobehav. Rev.* 45 100–118. 10.1016/j.neubiorev.2014.05.009 24875392

